# Lisosan G as a Modulator of Serum Lipid/Lipoprotein Changes, Lipid Metabolism and TGF-β1 Level in Neoplastic and Non-Neoplastic Liver Injury: A Rat Model Study

**DOI:** 10.3390/biology15030284

**Published:** 2026-02-05

**Authors:** Bartłomiej Szymczak, Luisa Pozzo, Szymon Zmorzyński, Anna Wilczyńska, Andrea Vornoli, Maria Lutnicka, Marta Wójcik

**Affiliations:** 1 Sub-Department of Pathophysiology, Department of Preclinical Veterinary Sciences, Faculty of Veterinary Medicine, University of Life Sciences in Lublin, Akademicka 12, 20-033 Lublin, Poland; 2 Institute of Agricultural Biology and Biotechnology, National Research Council, Via Moruzzi 1, 56124 Pisa, Italy; 3 Institute of Human Sciences, Faculty of Health Sciences, Academy of Zamość, Pereca Street 2, 22-400 Zamość, Poland; 4 Department of Epizootiology and Clinic of Infectious Diseases, Faculty of Veterinary Medicine, University of Life Sciences in Lublin, 20-612 Lublin, Poland; 5 Medical Faculty-Student, the J.P. II Catholic University of Lublin, 20-708 Lublin, Poland; 6 Oncology Lab, Department of Epizootiology and Clinic of Infectious Diseases, Faculty of Veterinary Medicine, University of Life Sciences in Lublin, 20-612 Lublin, Poland

**Keywords:** Lisosan G, hepatocellular carcinoma, lipid metabolism, nutraceuticals, lipoproteins

## Abstract

Chronic liver injury is accompanied by coordinated disturbances in lipid trafficking and inflammatory–fibrogenic signaling. Transforming growth factor beta 1 (TGF-β1) signaling has been implicated in hepatic fibrogenesis and tumor-associated remodeling and may co-vary with disturbances in lipid trafficking. Lisosan G (LG), a fermented wheat-derived nutraceutical, has reported antioxidant and anti-inflammatory activity and may influence these interconnected pathways. This study evaluated whether dietary LG alters the lipid composition of plasma lipoprotein fractions and hepatic TGF-β1 levels across distinct liver contexts. Seventy-two female Wistar rats were randomized into nine groups (*n* = 8/group) defined by liver condition, consisting of healthy control (Control), non-neoplastic liver (PH), and neoplastic liver injury (HCC; PH followed by diethylnitrosamine, DEN), and diet (standard diet, SD + 2.5% LG, or SD + 5% LG). Plasma lipoproteins (VLDL, LDL, HDL_1_, HDL_2_) were isolated by stepwise KBr density-gradient ultracentrifugation, and cholesterol (TC), phospholipids (PL), and triacylglycerols (TG) were quantified in each fraction. Hepatic TGF-β1 was measured by ELISA and normalized to total protein. LG effects depended strongly on baseline liver status, with significant Condition × Diet interactions for most lipid endpoints and for hepatic TGF-β1. In healthy rats, LG produced fraction-selective remodeling rather than uniform lipid lowering, including increased VLDL-TG at both doses and non-linear changes in cholesterol distribution across LDL and HDL subfractions. After PH, LG broadened lipid remodeling, including reduced VLDL-PL, increased VLDL-TG (both doses), and an increase in LDL-TC at 5% LG, accompanied by marked changes in HDL_1_/HDL_2_ cholesterol partitioning. In HCC, LG induced pronounced, often dose-dependent increases in LDL-associated lipids (LDL-PL, LDL-TG, LDL-TC) and increased HDL_1_-TC while decreasing HDL_2_-TC. Hepatic TGF-β1 was elevated in PH and further increased in HCC versus controls; LG reduced hepatic TGF-β1 in a condition-dependent manner, with the strongest reduction at 5% LG in HCC. Dietary Lisosan G remodels circulating lipoprotein lipid composition in a liver-status-dependent manner and is associated with reduced hepatic TGF-β1 abundance in injured liver, most prominently in neoplastic injury. These findings are consistent with the notion that nutraceutical interventions may show stronger phenotypic effects under perturbed metabolic–fibrogenic states than under stable physiology, while highlighting the need for mechanistic work to distinguish altered lipoprotein secretion from changes in peripheral clearance and to assess pathway-level TGF-β signaling.

## Simple Summary

Chronic liver injury disrupts blood lipid transport and activates inflammatory–fibrogenic signaling. Transforming growth factor β1 (TGF-β1) is a key mediator and Lisosan G (LG), a fermented wheat-derived nutraceutical, has been proposed to influence these processes. Seventy-two Wistar rats were assigned to healthy controls, with non-neoplastic (PH) or neoplastic liver injury (HCC; PH followed by diethylnitrosamine), and fed a standard diet or the same diet supplemented with 2.5% or 5% LG. Plasma lipoproteins (VLDL, LDL, HDL_1_, HDL_2_) were isolated to measure cholesterol, phospholipids, and triacylglycerols, and TGF-β1 in liver was quantified by ELISA. LG responses were liver-status-dependent. In healthy rats, LG caused selective changes, including higher VLDL triacylglycerols and non-linear changes in cholesterol distribution across LDL and HDL subfractions. After PH, LG lowered VLDL phospholipids, raised VLDL triacylglycerols, and increased LDL cholesterol at 5% LG, with marked changes in HDL_1_/HDL_2_ cholesterol partitioning. In HCC, LG produced the strongest remodeling, with dose-related increases in LDL-associated lipids, increased HDL_1_ cholesterol, and decreased HDL_2_ cholesterol. Hepatic TGF-β1 rose after PH and peaked in HCC; LG reduced it in injured liver, especially in HCC. Overall, LG supplementation was associated with liver-status-dependent changes in lipoprotein lipids and reduced hepatic TGF-β1 abundance in injured liver. Mechanistic studies are needed to distinguish effects on secretion versus clearance and to assess pathway-level TGF-β signaling.

## 1. Introduction

Liver diseases remain a major global health challenge, affecting both humans and companion animals. Chronic liver disorders are characterized by profound metabolic dysregulation, including disturbances in lipid metabolism and inflammatory signaling, which contribute to disease progression and impaired regeneration [1,2]. The liver is central to systemic lipid homeostasis, regulating the synthesis, transport, and degradation of triglycerides (TG), phospholipids (PL), and cholesterol through lipoprotein assembly and secretion [3,4]. Perturbations in these processes accompany a broad spectrum of hepatic pathologies, from transient injury following hepatectomy to malignant transformation in hepatocellular carcinoma (HCC) [5,6]. Partial hepatectomy induces an abrupt loss of functional liver mass, leading to transient suppression of lipoprotein synthesis and metabolic remodeling necessary for tissue regeneration [7]. Conversely, HCC development involves a chronic reprogramming of lipid metabolism that supports tumor proliferation and invasiveness through enhanced lipogenesis and altered lipid signaling [8,9]. Transforming growth factor beta (TGF-β) plays a pivotal role in these processes by coupling inflammation and fibrosis with lipid dysregulation. It inhibits the synthesis of apolipoprotein B-100 (apoB-100) and microsomal triglyceride transfer protein (MTP), thereby suppressing very-low-density lipoprotein (VLDL) assembly, while simultaneously promoting fibrogenesis and epithelial–mesenchymal transition [10,11,12]. Growing evidence indicates that nutraceuticals of plant origin may beneficially modulate these interconnected pathways. Lisosan G (LG), a fermented wheat-derived nutraceutical rich in polyphenols (ferulic and p-coumaric acid), vitamins, and unsaturated fatty acids, has demonstrated antioxidant, anti-inflammatory, and hepatoprotective properties [13,14]. Experimental studies have shown that LG activates Nrf2-dependent cytoprotective enzymes, suppresses NF-κB signaling, and improves serum lipid profiles by reducing triglycerides and LDL cholesterol [15,16]. Considering the close interdependence of lipid metabolism and TGF-β-mediated fibrogenic signaling, as well as the potential of natural dietary compounds to restore hepatic homeostasis, this study aimed to evaluate the effects of dietary supplementation with Lisosan G in three experimental rat models: healthy controls, rats after partial hepatectomy, and rats with chemically induced hepatocellular carcinoma (hepatectomy followed by DEN administration). The analysis focused on the lipid composition of plasma lipoprotein fractions, namely cholesterol, phospholipids, and triglycerides, and on hepatic TGF-β levels as a marker of pro-fibrogenic and signaling activity.

## 2. Materials and Methods

The present study was conducted on 72 female Wistar rats (6–8 weeks of age; body weight 180–200 g) obtained from the Center for Experimental Medicine at the Medical University of Lublin (Lublin, Poland). All procedures were approved by the Local Ethical Committee for Animal Experiments in Lublin (Approval No. 27/2023, Date 3 April 2023) and were carried out in accordance with the provisions of Directive 2010/63/EU on the protection of animals used for scientific purposes.

After a 7-day acclimatization period, animals were allocated to the nine experimental groups (*n* = 8 per group) using a computer-generated randomization sequence. The allocation list was prepared by an investigator not involved in outcome assessment and implemented using sequentially numbered animal IDs. Samples were coded to ensure blinding during analyses. The experimental design consisted of three main groups, representing different liver conditions: healthy (Control), partial hepatectomy (PH), and hepatocellular carcinoma (HCC). Each main group was further divided into three subgroups according to the type of diet: a standard diet (SD), a diet supplemented with 2.5% Lisosan G (LG2.5%), and a diet supplemented with 5% Lisosan G (LG5%). This resulted in the following experimental combinations: Control + SD, Control + LG2.5%, Control + LG5%; PH + SD, PH + LG2.5%, PH + LG5%; and HCC + SD, HCC + LG2.5%, HCC + LG5%. Rats received a standard complete diet for laboratory rodents (LSM; Agropol, Motycz, Poland) as the basal SD. Experimental diets were prepared from the same basal SD by incorporating Lisosan^®^ G (LG) at final concentrations of 2.5% (*w*/*w*) (LG2.5%) or 5% (*w*/*w*) (LG5%) by replacing an equivalent weight fraction of the basal SD, so that diets were formulated to be isoenergetic and differed only in LG content. Lisosan^®^ G was provided by Agrisan Srl (Larciano, Pistoia, Italy). Food intake and body weight were monitored throughout the experiment to verify comparable dietary exposure and to detect potential diet-related differences in caloric intake. Our previous studies [17,18] demonstrated that a sample size of *n* = 8 per experimental combination provides sufficient statistical power for comparable experimental designs. Rats assigned to the PH and HCC groups underwent two-thirds partial hepatectomy under general anesthesia (ketamine 90 mg/kg and xylazine 10 mg/kg, i.m.). A median laparotomy was performed, and the left lateral and right lobes of the liver were surgically excised. Animals were monitored intensively for seven postoperative days, with particular attention to behavior, food and water intake, and wound healing. Thereafter, they were monitored routinely until the end of the experiment. PH animals were euthanized 12 weeks after partial hepatectomy. In the HCC groups, following PH, diethylnitrosamine (DEN) was administered (50 mg/L in drinking water) continuously for 12 weeks, according to previously validated protocols [17,18]. This two-stage model has been shown to reliably induce hepatocellular carcinoma in rodents. At the end of the experimental period, all animals were re-anesthetized with ketamine/xylazine. Whole blood was collected by cardiac puncture into tubes containing ethylenediaminetetraacetic acid (EDTA) and processed immediately by centrifugation at 2500 rpm for 15–20 min at 4 °C to obtain plasma. Liver tissue samples were isolated, rinsed in ice-cold saline, snap-frozen in liquid nitrogen, and stored at −80 °C for subsequent analyses, including the determination of transforming growth factor β1 (TGF-β1) concentrations. To minimize observer bias, all samples were coded during biochemical and molecular analyses. Investigators performing outcome measurements were blinded to group assignments. Due to the nature of the surgical and carcinogenic interventions, blinding during the experimental procedures was not feasible.

### 2.1. Lipoprotein Fraction Isolation

**Figure 1:**
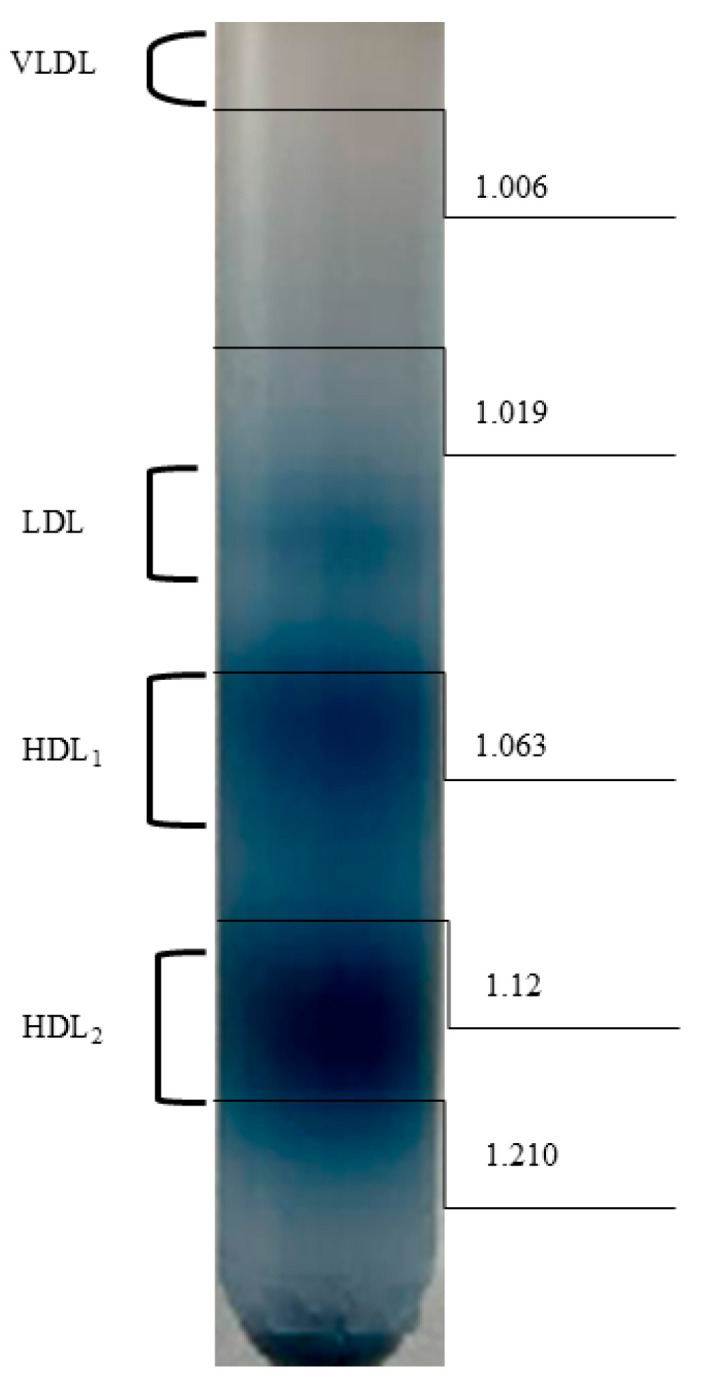
Schematic overview of rat plasma lipoprotein fractionation by stepwise potassium bromide (KBr) density-gradient ultracentrifugation. EDTA plasma was adjusted with KBr and layered under discontinuous KBr solutions spanning d = 1.006–1.21 g/mL, then centrifuged in a swinging-bucket rotor (Beckman SW 60-Ti). Band localization was guided using a Sudan Black-stained reference plasma processed in parallel.

Plasma lipoproteins were fractionated by density-gradient ultracentrifugation according to the method previously described by Eaton and Kipnis [19]. EDTA plasma was separated using stepwise KBr density-gradient ultracentrifugation with a swinging-bucket rotor (SW 60 Ti; Beckman Coulter, Brea, CA, USA in n Optima XPN-100 ultracentrifuge (Beckman Coulter, Brea, CA, USA). KBr solutions were prepared gravimetrically to final densities spanning 1.006–1.21 g/mL. For each sample, plasma (0.5 mL) was adjusted to the starting density (1.21 g/mL) with solid KBr and overlaid with stepwise KBr solutions from higher to lower density to form a discontinuous gradient. Tubes were centrifuged at 45,000 rpm (2.7 × 10^5^× *g* at rmax) at 10 °C for 24 h. Following centrifugation, lipoprotein bands were aspirated sequentially from the top of the tube using an 18 G needle (DispoFine; Zarys International Group, Zabrze, Poland), and fraction collection volumes were pre-defined and kept constant across all samples to minimize cross-contamination. Fractions were assigned according to buoyant-density cut-offs for rat plasma [20]: VLDL (d < 1.006 g/mL), LDL (d = 1.019–1.063 g/mL), HDL_1_ (d = 1.063–1.12 g/mL), and HDL_2_ (d = 1.12–1.21 g/mL). Band localization was guided by a reference plasma sample stained with Sudan Black run in parallel (Figure 1). Samples for further analysis were stored at −80 °C.

### 2.2. Phosphorus Concentration Measurement

Inorganic phosphorus was measured in lipoprotein fractions using the Fiske–Subbarow colorimetric method. To this end, 1 mL of the sample was mixed with 25% (*w*/*v*) ammonium molybdate ((NH_4_)_6_Mo_7_O_24_) solution in 2.5 M H_2_SO_4_ and 0.2% 1-amino-2-naphthol-4-sulfonic acid solution, along with 10% trichloroacetic acid. Ultra-pure water (Milli-Q, Merck Millipore/Merck KGaA, Darmstadt, Germany) was used for all analyses. Absorbance was measured at 660 nm (UV-VIS spectrophotometer, LAMBDA 365+; PerkinElmer, Shelton, CT, USA, 1 cm cuvette), using a control sample (0.5 mL H_2_O instead of the sample) as a blank. A standard solution containing a known concentration of phosphorus (phosphorus standard solution for AAS, 1 mg/mL P in water, Thermo Scientific, Thermo Fisher Scientific, Waltham, MA, USA) was used for calibration.

### 2.3. Cholesterol Determination

Cholesterol content in the lipoprotein fractions was determined by the Liebermann–Burchard method, adding 1 mL of sample to 1 mL of the reaction mixture, consisting of concentrated sulfuric acid, acetic anhydride, and glacial acetic acid. The test tubes were incubated for 10 min at 25 °C, protected from light. Absorbance was measured at 500 nm using a UV-VIS spectrophotometer (LAMBDA 365+; PerkinElmer, Shelton, CT, USA) with a 1 cm optical path cuvette. A mixture of 1 mL of water and 1 mL of reagent was used as the blank. Cholesterol standard solutions were prepared in the range of 50–400 mg/dL by serial dilution, and cholesterol concentrations in the samples were calculated based on the standard curve.

### 2.4. Triglyceride Measurement

Triglyceride concentrations in the lipoprotein fractions were determined using the commercially available Liquick Cor-TG 30 kit (PZ Cormay S.A., Warsaw, Poland)). Triglyceride concentrations were measured according to the manufacturer’s instructions.

### 2.5. Liver Homogenate Preparation and TGF-β1 Measurement

Liver tissue was carefully cleaned of connective and adipose tissue, then homogenized in cold buffer (containing Roche complete protease inhibitor and EDTA) to obtain a uniform suspension. The homogenate was centrifuged at 4 °C for 10 min at 10,000× *g* to remove insoluble cell debris. The supernatant was filtered through a syringe filter (PVDF) and was used for subsequent protein concentration determination and cytokine analysis. TGF-β1 levels in the liver homogenate were measured using the TGF-β1 ELISA Kit (ADI-900-155, Enzo Life Sciences, Enzo Life Sciences, Farmingdale, NY, USA) according to the manufacturer’s instructions. Briefly, aliquots of each sample were acidified with HCl, incubated for 10 min at room temperature, and subsequently neutralized with NaOH/HEPES to pH 7.2–7.6. Activated samples were then diluted with the sample diluent and assayed immediately. Kit standards (active recombinant TGF-β1) were not acid-activated. The plates were measured at a wavelength of 450 nm using a ELx800 (BioTek Instruments, Winooski, VT, USA) plate reader. All measurements were performed in triplicates for each sample. To ensure the reliability of the results, control samples and blanks were included in each measurement. Total protein was quantified in the same clarified liver homogenate supernatants used for ELISA. TGF-β1 values were normalized to protein content as TGF-β1 (pg/mg protein) = [TGF-β1] (pg/mL)/[total protein] (mg/mL), with both terms corrected for all sample dilution factors.

Total protein concentration in liver homogenates was determined using the Lowry method, as modified by Peterson. Briefly, aliquots of clarified liver homogenate supernatants were precipitated with trichloroacetic acid, centrifuged to obtain a protein pellet, and the pellet was subsequently re-dissolved in alkaline solution prior to color development. Thereafter, 100 µL of the re-solubilized sample or protein standard (bovine serum albumin, BSA; 0–500 µg/mL; standards prepared in the same buffer as samples) was mixed with 1 mL of alkaline copper reagent (reagent A; 2% Na_2_CO_3_ in 0.1 M NaOH containing 1% CuSO_4_·5H_2_O and 2% KNaC_4_H_4_O_6_·4H_2_O mixed in a 100:1:1 ratio). After 10 min incubation at room temperature, 100 µL of Folin–Ciocalteu phenol reagent (diluted 1:1 with distilled water immediately before use) was added rapidly, mixed, and incubated for 30 min at room temperature in the dark. Absorbance was measured at 750 nm using a UV–VIS spectrophotometer (PerkinElmer Lambda 365+, 1 cm path length). All samples and standards were analyzed in triplicate. Protein concentrations were calculated from the BSA standard curve and corrected for all dilution factors. Blank samples containing the homogenization buffer processed in parallel (including the precipitation/resolubilization steps) were included for baseline correction.

### 2.6. Histopatological Examination

Liver samples were fixed in 10% phosphate-buffered formalin for 24 h at room temperature, processed routinely, embedded in paraffin, and sectioned at 3–5 µm using a rotary microtome (Leica SR-200; Leica Microsystems, Milton Keynes, UK). Sections were stained with hematoxylin (5 min) and eosin (5 min) (H&E) at room temperature. Histological evaluation was performed using a light microscope (Eclipse E-600; Nikon, Tokyo, Japan) at ×200 magnification. Hepatic lesions were assessed in accordance with the World Health Organization (WHO) histological classification of tumors.

### 2.7. Statistical Analysis

The individual animal was the experimental unit (*n* = 8 per group). For each outcome, three technical replicates were averaged to obtain a single value per animal prior to inferential statistics. Data are presented as mean ± standard deviation (SD). For each lipid outcome and hepatic TGF-β1, effects of liver condition (Control, PH, HCC), diet (SD, LG2.5%, LG5%), and their interaction were assessed using a two-way analysis of variance (ANOVA) including the Condition × Diet interaction term. Because the primary objective was to quantify diet-related contrasts within each liver condition (Control, PH, HCC), pairwise comparisons among diets were performed as simple effects within each condition for every outcome. Specifically, within each condition, we compared Standard Diet vs. LG 2.5%, Standard Diet vs. LG 5%, and LG 2.5% vs. LG 5%, and adjusted these within-condition comparisons using Tukey’s honestly significant difference (HSD) procedure (family-wise α = 0.05). Although interpretation primarily emphasized conditions/outcomes, showing a significant Condition × Diet interaction in the two-way ANOVA, within-condition diet contrasts are presented for completeness across endpoints. Model assumptions were evaluated using visual inspection of residual plots and Shapiro–Wilk tests for normality of residuals, together with Levene’s (Brown–Forsythe) tests for homogeneity of variances across groups. Effect sizes are reported as partial eta-squared (ηp^2^) for main effects and interactions, together with 95% confidence intervals (95% CI) calculated using the noncentral F distribution. Associations between hepatic TGF-β1 and lipoprotein lipid measures were explored using Pearson’s correlation coefficients (r) within each Condition × Diet subgroup. Statistical significance was set at *p* < 0.05 (two-sided). All analyses were performed in STATISTICA 13.3 (TIBCO, Palo Alto, CA, USA) and GraphPad Prism 8 (GraphPad Software, San Diego, CA, USA).

## 3. Results

Across lipoprotein lipid endpoints, two-way ANOVA demonstrated that the impact of LG depended on baseline liver status, supporting the interpretation of dietary contrasts within each condition (Control, PH, HCC). For plasma lipids, the main effect of Condition was uniformly large across fractions (ηp^2^ ≈ 0.82–0.98 for most endpoints), and Condition × Diet interactions were most pronounced for cholesterol measures, including LDL-TC (ηp^2^ = 0.8241, 95% CI [0.7894, 0.8481]), HDL_1_-TC (ηp^2^ = 0.6696 [0.6172, 0.7071]), and HDL_2_-TC (ηp^2^ = 0.8864 [0.8669, 0.8998]). Interactions were also evident for triglycerides in selected fractions (VLDL-TG: ηp^2^ = 0.2514 [0.1217, 0.3455]; HDL_1_-TG: ηp^2^ = 0.4135 [0.3299, 0.4786]; HDL_2_-TG: ηp^2^ = 0.3885 [0.3003, 0.4541]). For phospholipids, interactions were frac-ion-dependent, with moderate effects for VLDL-PL (ηp^2^ = 0.1920 [0.0517, 0.3027]) and LDL-PL (ηp^2^ = 0.2896 [0.1685, 0.3860]), but negligible for HDL_2_-PL (ηp^2^ = 0.0008). Overall, diet-related differences were fraction- and lipid-class-selective rather than uniformly lipid-lowering.

In healthy rats, LG did not significantly affect phospholipids in any fraction. In contrast, triglyceride and cholesterol endpoints showed selective changes (Table 1). The most consistent triglyceride effect was an increase in VLDL-TG at both LG doses, whereas LDL-TG and HDL_1_-TG did not differ from SD but differed between LG doses; HDL_2_-TG showed at most a trend versus SD at 5% LG (*p* = 0.0529). For cholesterol, LG increased VLDL-TC versus SD at both doses. LDL-TC decreased only at 2.5% LG, with no significant SD versus 5% LG contrast, and the two LG doses differed (*p* = 0.0143). HDL cholesterol displayed a non-linear dose pattern: HDL_1_-TC decreased at 2.5% LG but increased at 5% LG, while HDL_2_-TC was reduced at 2.5% LG but not significantly changed at 5% LG.

Following partial hepatectomy, lipid profiles differed from controls and LG effects broadened (Table 1). For phospholipids, VLDL-PL was reduced under both LG doses, whereas LDL-PL and HDL-PL were unchanged. For triglycerides, VLDL-TG increased at both LG doses; LDL-TG increased at 5% LG; and HDL_2_-TG was higher under both LG doses. For cholesterol, VLDL-TC did not differ across diets, but LDL-TC increased markedly at 5% LG. HDL fractions were strongly diet-sensitive in PH: HDL_1_-TC and HDL_2_-TC differed across all pairwise comparisons, with the largest reduction observed for HDL_2_-TC at 2.5% LG.

In HCC, baseline circulating lipids were markedly altered and LG supplementation produced strong, frequently dose-dependent shifts (Table 1). For phospholipids, VLDL-PL did not differ significantly between SD and either LG dose, but the two LG doses differed (*p* = 0.0116); LDL-PL increased with LG. For triglycerides, VLDL-TG showed a clear high-dose effect (SD versus 2.5% LG not significant; 5% LG increased markedly and differed from 2.5% LG). LDL-TG increased under both LG doses. HDL_1_-TG and HDL_2_-TG increased strongly under LG (both SD contrasts *p* < 0.0001), with a dose difference for HDL_1_-TG but not HDL_2_-TG. For cholesterol, VLDL-TC remained unchanged, whereas LDL-TC increased dramatically and dose-dependently; HDL_1_-TC increased while HDL_2_-TC decreased.

**Figure 2:**
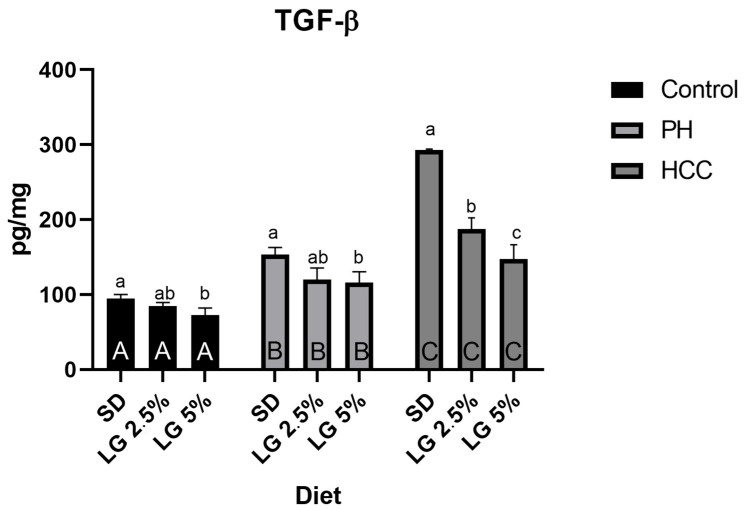
Hepatic TGF-β levels. Data are presented as mean ± SD. Different letters indicate statistically significant differences determined by two-way ANOVA. Uppercase letters denote significant differences (*p* ≤ 0.05) between main groups under the same diet, whereas lowercase letters indicate differences among diets within the same main group. SD—standard diet; LG 2.5%—diet supplemented with 2.5% Lisosan G; LG 5%—diet supplemented with 5% Lisosan G; PH—partial hepatectomy; HCC—hepatocellular carcinoma.

Hepatic TGF-β1 concentrations were significantly elevated in PH and HCC relative to controls (94.4 ± 5.7 pg/mg protein), reaching 153.7 ± 9.3 pg/mg in PH and 292.7 ± 1.5 pg/mg in HCC. LG reduced hepatic TGF-β1 in a condition-dependent manner, reflected by a significant Condition × Diet interaction (F (4,63) = 68.81, *p* < 0.0001, ηp^2^ = 0.814). In PH, 5% LG reduced TGF-β1 to 115.8 ± 14.6 pg/mg (*p* < 0.01 vs. PH), while in HCC the same dose reduced it to 147.5 ± 18.9 pg/mg (*p* < 0.0001 vs. HCC) (Figure 2).

Pearson correlations were assessed within Condition × Diet subgroups (*n* = 8) to examine associations between hepatic TGF-β1 and selected lipid measures. In controls, phospholipid correlations were generally weak and non-significant, whereas several associations involving triglycerides and total cholesterol reached significance. In PH and HCC, positive correlations between TGF-β1 and selected lipid parameters were more frequently observed, particularly for phospholipids and triglycerides in VLDL and LDL fractions. Given the small subgroup sample size and the number of tested associations, these correlations are reported as exploratory patterns of co-variation rather than evidence of mechanistic relationships.

**Figure 3:**
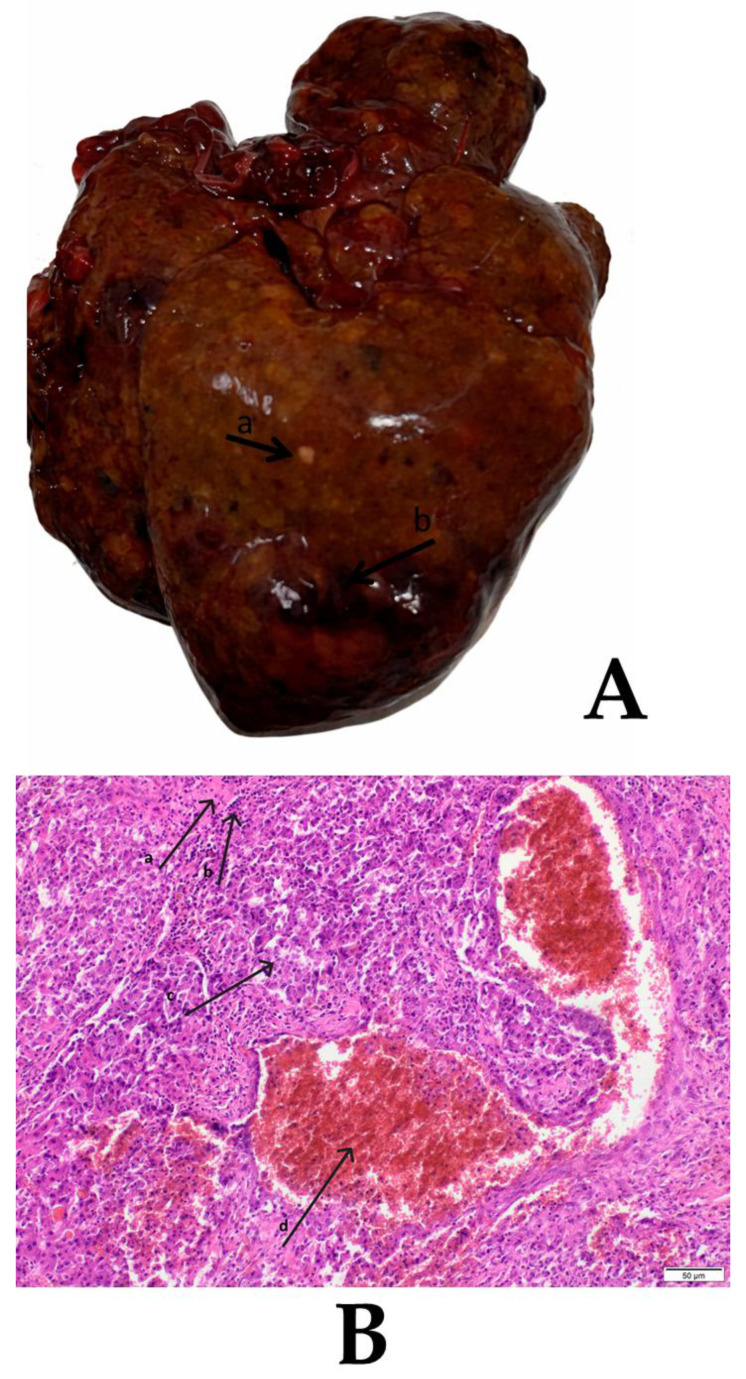
Macroscopic and histopathological view of hepatocellular carcinoma (HCC). (**A**) Representative gross appearance of livers collected from the HCC group, showing visible tumor-associated alterations in the hepatic surface and parenchyma. Black arrows indicate (a) pale areas consistent with focal parenchymal alteration and (b) regions of hemorrhage/blood extravasation. (**B**) Representative H&E-stained liver section from the HCC group. Black arrows indicate areas of extensive hemorrhage and necrosis within the tumor mass. Marked regions: a, necrotic focus; b, region of inflammatory infiltration; c, region of atypical hepatocytes; d, hemorrhage/extravasation (blood extravasation).

On gross examination, livers from the PH group appeared normal, with a smooth capsular surface, uniform reddish-brown coloration, and preserved lobar architecture without visible focal lesions. In contrast, livers from the HCC group showed features consistent with chronic injury, including mild-to-moderate enlargement and a heterogeneous surface with multifocal discoloration. Histologically, PH livers exhibited preserved hepatic architecture with orderly hepatocyte plates and sinusoidal spaces and no inflammatory infiltrates or cytological abnormalities. HCC sections showed chronic injury with multifocal areas of cellular atypia (Figure 3), characterized by lymphocytic infiltration and hepatocellular alterations, including enlarged nuclei, increased nuclear-to-cytoplasmic ratio, and pleomorphic nuclear morphology, within areas of parenchymal injury consistent with neoplastic remodeling in DEN-exposed livers.

## 4. Discussion

The study evaluated whether LG supplementation modulates the lipid composition of major plasma lipoprotein fractions and hepatic TGF-β1 concentrations across three bio-logical contexts: healthy rats (Control), rats with non-neoplastic liver injury following partial hepatectomy (PH), and rats with neoplastic liver injury (HCC) induced in a PH + DEN model. A central finding was the presence of significant Condition × Diet interactions for most lipid endpoints and for hepatic TGF-β1, indicating that the effects of LG were con-tingent on baseline liver status. Consequently, LG-related contrasts are best interpreted within each condition (Control, PH, and HCC) rather than pooled across contexts. Across injured-liver contexts (PH and HCC), LG was associated with broader and fraction-dependent remodeling of lipoprotein lipid composition and with lower hepatic TGF-β1 (most clearly at the 5% dose), whereas in healthy animals its effects were smaller and more fraction-selective. This pattern is consistent with nutraceutical interventions whose phenotypic impact is typically more pronounced when metabolic and inflammatry–fibrogenic networks are perturbed rather than under stable physiology [15,21,22]. A key translational limitation is that rodent lipoprotein biology differs from that of humans, which constrains direct clinical inference from HDL subfraction changes and from the partitioning of lipids across lipoprotein classes [23,24]. Against this background, the Control group provides a useful reference for the direction and dose-dependence of LG effects under baseline conditions. In healthy rats, LG induced moderate, fraction-specific alterations rather than a uniform lipid-lowering profile. The most consistent change was an increase in VLDL-TG at both LG doses, while cholesterol-related endpoints showed a non-linear dose–response (e.g., LDL-TC decreased at 2.5% LG without a comparable effect at 5%). Such non-linearity is common for complex nutraceutical mixtures and argues against a single dominant mechanism uniformly governing all lipoprotein fractions [22,25]. The observed fraction and dose-specific pattern is compatible with changes in lipid fluxes involving hepatic secretion, peripheral clearance, and/or interfraction remodeling; however, the present dataset does not allow these processes to be disentangled [26,27]. Several non-mutually exclusive mechanisms remain plausible but cannot be resolved from the current dataset. One possible explanation is that bioactive constituents of fermented wheat-derived products (including polyphenols and other antioxidant compounds) may influence hepatic fatty-acid uptake and oxidation, thereby affecting the availability of triacylglycerols for VLDL assembly, but this was not directly assessed in the present study [28,29]. In principle, depending on the balance between β-oxidation and esterification, VLDL-TG may increase without a parallel rise in LDL-cholesterol. Hepatic VLDL output is tightly regulated, including insulin-dependent post-translational control of apoB stability and the activity of MTP [30,31,32]. As apoB/apoA-I levels, MTP biology, and indices of lipoprotein clearance were not measured, the data cannot distinguish increased VLDL production from reduced peripheral catabolism, an important limitation when interpreting VLDL-TG changes. For hepatic TGF-β1, baseline concentrations in healthy liver are expected to be relatively low, providing limited dynamic range for further nutraceutical-associated reductions [33,34]. Accordingly, the lack of a marked LG effect on hepatic TGF-β1 in Control is biologically plausible and consistent with the view that anti-inflammatory and antioxidant actions of nutraceuticals are most evident under conditions of stress or tissue injury [35,36]. Following PH, hepatocellular priorities shift toward proliferation, membrane biogenesis, and energy generation. Liver regeneration is commonly accompanied by transient hepatic lipid accumulation, reflecting coordinated changes in lipid uptake, *De Novo* lipogenesis, lipid-droplet dynamics, and a temporary suppression of lipid export via apoB-containing lipoproteins [37,38]. In this context, the reduced plasma concentrations observed under the standard diet in PH (e.g., lower VLDL-PL and LDL-TC) are consistent with a transient state in which lipid resources are preferentially retained and utilized locally within the regenerating liver rather than exported to the circulation [39,40]. In PH, the LG-associated changes, most notably the increase in VLDL-TG at both doses and the increase in LDL-TC at 5%, may be consistent with condition-specific remodeling of lipid partitioning during regeneration; however, secretion versus clearance cannot be inferred without kinetic measures and markers of apoB-dependent export [41,42]. However, without direct markers of secretion versus clearance and without parallel measures of liver injury severity, causal attribution remains uncertain. In the HCC context, animals on the standard diet exhibited markedly altered and, in several fractions, reduced circulating VLDL-TG and LDL-TC relative to the other conditions. Local lipid synthesis and accumulation within the tumor tissue may increase despite reduced systemic availability of circulating lipoproteins; however, tumor lipid content, *De Novo* lipogenesis, and lipid uptake were not quantified in the present study [43,44]. HCC cells may intensify *De Novo* lipogenesis and lipid uptake to support proliferation, while the diseased liver may exhibit impaired capacity to assemble and export apoB-containing lipoproteins. Advanced neoplastic transformation may disrupt lobular architecture and reduce the pool of metabolically competent hepatocytes, which could limit VLDL production/secretion and secondarily reduce LDL-cholesterol and LDL-cholesterol generation. Moreover, cirrhosis and severe chronic liver disease are often accompanied by hypolipidemia due to diminished hepatic synthetic function [45,46]. In this setting, the increases in VLDL-TG and LDL-TC observed after LG supplementation should be interpreted cautiously, as they could reflect altered hepatic lipid handling and/or peripheral clearance, and may also be influenced by between-group differences in injury severity or neoplastic burden [47,48]. Hepatic TGF-β1 was elevated in PH and further increased in HCC, consistent with the established involvement of TGF-β signaling in hepatic injury responses, fibrogenesis, and neoplastic progression, including processes such as EMT [33,49,50]. LG lowered hepatic TGF-β1 in a condition-dependent manner, most prominently in the neoplastic context. At the same time, it should be emphasized that the present work quantified hepatic TGF-β1 protein levels and did not assess pathway activation or downstream fibrogenic readouts; thus, the findings support an association with lower TGF-β1 abundance rather than definitive suppression of TGF-β signaling. Correlations between TGF-β1 and lipid parameters are therefore best regarded as exploratory, given the limited subgroup sample size, multiple testing burden, and the inability to infer causal directionality. Only female Wistar rats were used; therefore, the findings cannot be directly generalized to males. This is particularly relevant for DEN-based hepatocarcinogenesis, where marked sex differences have been reported, with estrogen-dependent suppression of Kupffer cell IL-6 responses proposed to contribute to reduced susceptibility of females to DEN-driven liver tumorigenesis [51]. The PH component adds an additional layer of sexual dimorphism because estradiol and estrous-cycle stage modulate hepatocyte proliferation and regeneration kinetics after partial hepatectomy [52]. Moreover, fibrogenic signaling (including TGF-β1-linked pathways) and lipid metabolism are influenced by sex hormones; ovariectomy and estrogen-receptor modulation have been shown to alter DEN-associated injury and fibrotic phenotypes, indicating that hormonal milieu may confound baseline values and dietary responsiveness in female-only designs [53,54,55]. Future studies should include both sexes or explicitly control for estrous-cycle stage to test whether LG effects on hepatic TGF-β1 and lipoprotein lipid composition are sex-dependent [56].

## 5. Conclusions

In conclusion, Lisosan G supplementation was associated with liver status-dependent changes in the lipid composition of plasma lipoprotein fractions and in hepatic TGF-β1 levels, with the strongest and most widespread shifts observed in the injury models (PH and particularly HCC). The reduction in hepatic TGF-β1 abundance in the injured liver, occurring alongside remodeling of lipoprotein lipid composition, suggests that Lisosan G may influence markers related to metabolic and fibrogenic processes; however, pathway-level effects and causality cannot be established from the present data. At the same time, the lipid changes did not reflect a uniformly hypolipidemic pattern; instead, they indicated context- and fraction-dependent shifts in lipid partitioning among lipoprotein classes. Because the present data do not distinguish effects on hepatic secretion (apoB/MTP-dependent output), intravascular remodeling, and peripheral clearance, and because TGF-β pathway activity (e.g., downstream signaling and fibrogenic readouts) was not assessed, further mechanistic studies are required to clarify the mechanisms underlying these associations

## Figures and Tables

**Table 1:** Lipid composition of rat plasma. Data are mean ± SD of eight rats per group. Concentrations of lipid components is mg/dL.

Control					Statistical Significance (P)		
Diet		Standard Diet	LG 2.5%	LG 5%	SD. vs. LG 2.5%	SD. vs. LG 5%	LG 2.5% vs. 5%
Phospholipids							
	VLDL	12.74 ± 0.78	12.75 ± 1.02	12.55 ± 1.07	0.9992	0.9133	0.8973
	LDL	15.65 ± 1.53	15.11 ± 1.16	14.81 ± 1.21	0.8973	0.1930	0.8042
	HDL_1_	23.12 ± 0.83	23.12 ± 0.97	23.07 ± 0.93	>0.9999	>0.9999	>0.9999
	HDL_2_	20.80 ± 0.95	20.38 ± 1.37	20.76 ± 1.22	>0.9999	>0.9999	>0.9999
Triacylglycerols							
	VLDL	84.34 ± 6.76	91.95 ± 6.32	91.80 ± 10.39	0.0438	0.0490	0.9987
	LDL	4.66 ± 0.99	3.93 ± 1.08	5.03 ± 0.95	0.2305	0.6820	0.0402
	HDL_1_	2.79 ± 0.32	2.48 ± 0.37	3.12 ± 0.59	0.2172	0.1540	0.0020
	HDL_2_	1.45 ± 0.42	1.22 ± 0.29	1.15 ± 0.28	0.1768	0.0529	0.8349
Total cholesterol							
	VLDL	2.74 ± 0.36	3.22 ± 0.35	3.46 ± 0.39	0.0001	<0.0001	0.0973
	LDL	65.21 ± 2.89	59.01 ± 2.06	62.92 ± 1.92	<0.0001	0.2179	0.0143
	HDL_1_	108.58 ± 8.26	99.00 ± 3.45	117.35 ± 5.86	0.0002	0.0005	<0.0001
	HDL_2_	90.83 ± 2.41	80.13 ± 2.67	87.85 ± 2.29	<0.0001	0.0988	<0.0001
Partial hepatectomy (PH)					Statistical significance (P)		
Diet		Standard Diet	LG 2.5%	LG 5%	SD. vs. LG 2.5%	SD. vs. LG 5%	LG 2.5% vs. 5%
Phospholipids							
	VLDL	7.12 ± 1.77	5.79 ± 0.71	5.76 ± 0.79	0.0161	0.0129	0.9963
	LDL	7.72 ± 1.03	7.51 ± 0.95	7.08 ± 0.80	0.8904	0.3718	0.6465
	HDL_1_	19.85 ± 1.64	19.45 ± 1.03	18.94 ± 1.73	>0.9999	>0.9999	>0.9999
	HDL_2_	17.01 ± 1.44	16.60 ± 1.41	16.87 ± 1.70	>0.9999	>0.9999	>0.9999
Triacylglycerols							
	VLDL	49.52 ± 8.73	58.55 ± 3.71	62.41 ± 7.16	0.0135	0.0003	0.4315
	LDL	2.56 ± 1.07	3.58 ± 1.18	4.03 ± 1.12	0.0646	0.0042	0.5583
	HDL_1_	1.97 ± 0.34	2.42 ± 0.07	2.51 ± 0.50	0.0987	0.0107	0.6404
	HDL_2_	0.84 ± 0.21	1.18 ± 0.30	1.18 ± 0.31	0.0304	0.0285	0.9996
Total cholesterol							
	VLDL	0.92 ± 0.10	0.90 ± 0.08	0.90 ± 0.11	0.9887	0.9874	>0.9999
	LDL	47.21 ± 4.95	50.05 ± 2.96	59.17 ± 3.27	0.0992	<0.0001	<0.0001
	HDL_1_	124.36 ± 3.01	111.35 ± 2.62	151.46 ± 4.01	<0.0001	<0.0001	<0.0001
	HDL_2_	111.58 ± 2.73	76.06 ± 0.90	94.17 ± 2.08	<0.0001	<0.0001	<0.0001
Hepatocellular carcinoma (HCC)					Statistical significance (P)		
Diet		Standard Diet	LG 2.5%	LG 5%	SD. vs. LG 2.5%	SD. vs. LG 5%	LG 2.5% vs. 5%
Phospholipids							
	VLDL	4.20 ± 0.38	3.66 ± 0.37	5.04 ± 0.66	0.4752	0.1787	0.0116
	LDL	3.12 ± 0.17	4.34 ± 0.49	5.31 ± 0.31	0.0326	<0.0001	0.1107
	HDL_1_	12.03 ± 0.64	11.51 ± 0.44	12.60 ± 0.79	>0.9999	>0.9999	>0.9999
	HDL_2_	14.85 ± 1.19	14.34 ± 0.44	14.80 ± 1.13	>0.9999	>0.9999	>0.9999
Triacylglycerols							
	VLDL	20.52 ± 1.41	24.91 ± 1.41	42.85 ± 2.65	0.3385	<0.0001	<0.0001
	LDL	1.41 ± 0.17	2.48 ± 0.20	3.09 ± 0.29	0.0443	0.0008	0.3509
	HDL_1_	0.16 ± 0.04	1.19 ± 0.13	1.87 ± 0.13	<0.0001	<0.0001	0.0010
	HDL_2_	0.14 ± 0.02	0.81 ± 0.05	0.88 ± 0.13	<0.0001	<0.0001	0.8334
Total cholesterol							
	VLDL	0.76 ± 0.06	0.75 ± 0.04	0.75 ± 0.08	0.9972	0.9982	0.9999
	LDL	7.60 ± 0.54	19.59 ± 1.07	38.05 ± 2.08	<0.0001	<0.0001	<0.0001
	HDL_1_	57.93 ± 2.43	69.42 ± 2.90	88.13 ± 3.77	<0.0001	<0.0001	<0.0001
	HDL_2_	115.55 ± 5.28	88.81 ± 3.07	76.02 ± 2.15	<0.0001	<0.0001	<0.0001

## Data Availability

The data that support the findings of this study are available from the corresponding author upon reasonable request.

## Abbreviations

The following abbreviations are used in this manuscript:

TG	Triglycerides
PL	Phospholipids
TC	Total cholesterol
PH	Partial hepatectomy
HCC	Hepatocellular carcinoma
TGF-β	Transforming growth factor beta
apoB-100	Apolipoprotein B-100
MTP	Microsomal triglyceride transfer protein
VLDL	Very-low-density lipoprotein
LDL	Low-density lipoprotein
HDL	High-density lipoprotein
HDL_1_	High-density lipoprotein, subfraction 1
HDL_2_	High-density lipoprotein, subfraction 2
LG	Lisosan G
LG2.5%	Diet supplemented with 2.5% Lisosan G
LG5%	Diet supplemented with 5% Lisosan G
DEN	Diethylnitrosamine
EDTA	Ethylenediaminetetraacetic acid
BSA	Bovine serum albumin
ELISA	Enzyme-linked immunosorbent assay
SD	Standard diet
ANOVA	Analysis of variance
HSD	Tukey’s honestly significant difference
Nrf2	Nuclear factor erythroid 2-related factor 2
NF-κB	Nuclear factor kappa-light-chain-enhancer of activated B cells
CPT1A	Carnitine palmitoyltransferase 1A
UV-VIS	Ultraviolet–visible (spectrophotometry)
